# On the implementation of acollinearity in PET Monte Carlo simulations

**DOI:** 10.1088/1361-6560/ad70f1

**Published:** 2024-09-10

**Authors:** Maxime Toussaint, Francis Loignon-Houle, Étienne Auger, Gabriel Lapointe, Jean-Pierre Dussault, Roger Lecomte

**Affiliations:** 1 Sherbrooke Molecular Imaging Center of CRCHUS and Department of Nuclear Medicine and Radiobiology, Sherbrooke, Québec, Canada; 2 Instituto de Instrumentación para Imagen Molecular (I3M), Centro Mixto CSIC—Universitat Politécnica de Valéncia, Valencia, Spain; 3 IR&T Inc., Sherbrooke, Québec, Canada; 4 Independent researcher, Shefford, Québec, Canada; 5 Department of Computer Science, Université de Sherbrooke, Sherbrooke, Québec, Canada

**Keywords:** positron emission tomography, acollinearity, Monte Carlo simulation, spatial resolution

## Abstract

**Objective.:**

Acollinearity of annihilation photons (APA) introduces spatial blur in positron emission tomography (PET) imaging. This phenomenon increases proportionally with the scanner diameter and it has been shown to follow a Gaussian distribution. This last statement can be interpreted in two ways: the magnitude of the acollinearity angle, or the angular deviation of annihilation photons from perfect collinearity. As the former constitutes the partial integral of the latter, a misinterpretation could have significant consequences on the resulting spatial blurring. Previous research investigating the impact of APA in PET imaging has assumed the Gaussian nature of its angular deviation, which is consistent with experimental results. However, a comprehensive analysis of several simulation software packages for PET data acquisition revealed that the magnitude of APA was implemented as a Gaussian distribution.

**Approach.:**

We quantified the impact of this misinterpretation of APA by comparing simulations obtained with GATE, which is one of these simulation programs, to an in-house modification of GATE that models APA deviation as following a Gaussian distribution.

**Main results.:**

We show that the APA misinterpretation not only alters the spatial blurring profile in image space, but also considerably underestimates the impact of APA on spatial resolution. For an ideal PET scanner with a diameter of 81 cm, the APA point source response simulated under the first interpretation has a cusp shape with 0.4 mm FWHM. This is significantly different from the expected Gaussian point source response of 2.1 mm FWHM reproduced under the second interpretation.

**Significance.:**

Although this misinterpretation has been found in several PET simulation tools, it has had a limited impact on the simulated spatial resolution of current PET scanners due to its small magnitude relative to the other factors. However, the inaccuracy it introduces in estimating the overall spatial resolution of PET scanners will increase as the performance of newer devices improves.

## 1. Introduction

Annihilation photon acollinearity (APA) is a source of spatial blur in positron emission tomography (PET) imaging (Cherry *et al* 2003, Moses 2011). Due to its angular nature, the size of the blur induced in PET imaging is proportional to the size of the scanner (Levin and Hoffman 1999). In whole-body PET scanners, APA introduces an additional contribution of ≈2 mm in quadrature to the other factors affecting spatial resolution (Shibuya *et al* 2007, Moses 2011). Unlike detector size and detector multiplexing, acollinearity, like positron range, is a fundamental source of spatial blur that is more difficult to circumvent (Moses 2011).

Although some preliminary results suggest that ultra-high TOF resolution may eventually reduce the blur induced by APA (Toussaint *et al* 2020a), it remains important to understand and accurately model its effect on current and future scanners.

In PET, APA is described as following a Gaussian distribution (Harrison *et al* 1999, Levin and Hoffman 1999). This statement is based on studies of APA in positronium performed for a variety of materials, using setups relying on collimation and distance (DeBenedetti *et al* 1950, Lang and DeBenedetti 1957, Colombino *et al* 1960, 1963, 1964). These experiments required a lot of space and long acquisition times to obtain the necessary angular resolution and statistics. They have shown that APA follows a Gaussian distribution with a FWHM of ≈0.50° in water at 20 °C–30 °C. More recently, a different approach based on the variation of annihilation photon energy induced by acollinearity has been proposed to lessen the experimental burden of the previous studies (Shibuya *et al* 2007). This approach assumes that acollinearity is invariant with respect to the medium of interest, which is adequate for PET. Their results indicate that APA in a human subject follows a double Gaussian distribution with a combined FWHM of ≈0.55°.

The statement ‘APA follows a Gaussian distribution’ allows two interpretations: its *magnitude*, or its 2D angular *deviation* relative to the collinear case. Since the *magnitude* distribution is actually the partial integration of the *deviation* distribution, a misinterpretation gives rise to a markedly different profile. In fact, the first interpretation would mean that the acollinearity *deviation* follows a 2D Gaussian distribution divided by the norm of its argument, which turns out to be significantly sharper than a Gaussian distribution.

The blur induced by APA in the image space is described as having a Gaussian shape (Levin and Hoffman 1999), which corresponds to its *deviation* following a 2D Gaussian distribution. However, it appears that simulation software used for PET imaging, including GATE (Sarrut *et al* 2021), SimSET (Poon *et al* 2015), gPET (Lai *et al* 2019), URT-MCS (He *et al* 2023), and possibly others, simulate APA such that its *magnitude* follows a Gaussian distribution. In this Note, we demonstrate, mostly via GATE (Sarrut *et al* 2021), how this misinterpretation of the APA distribution underestimates the spatial blur induced in the image space.

## 2. Theory

**Figure 1.**
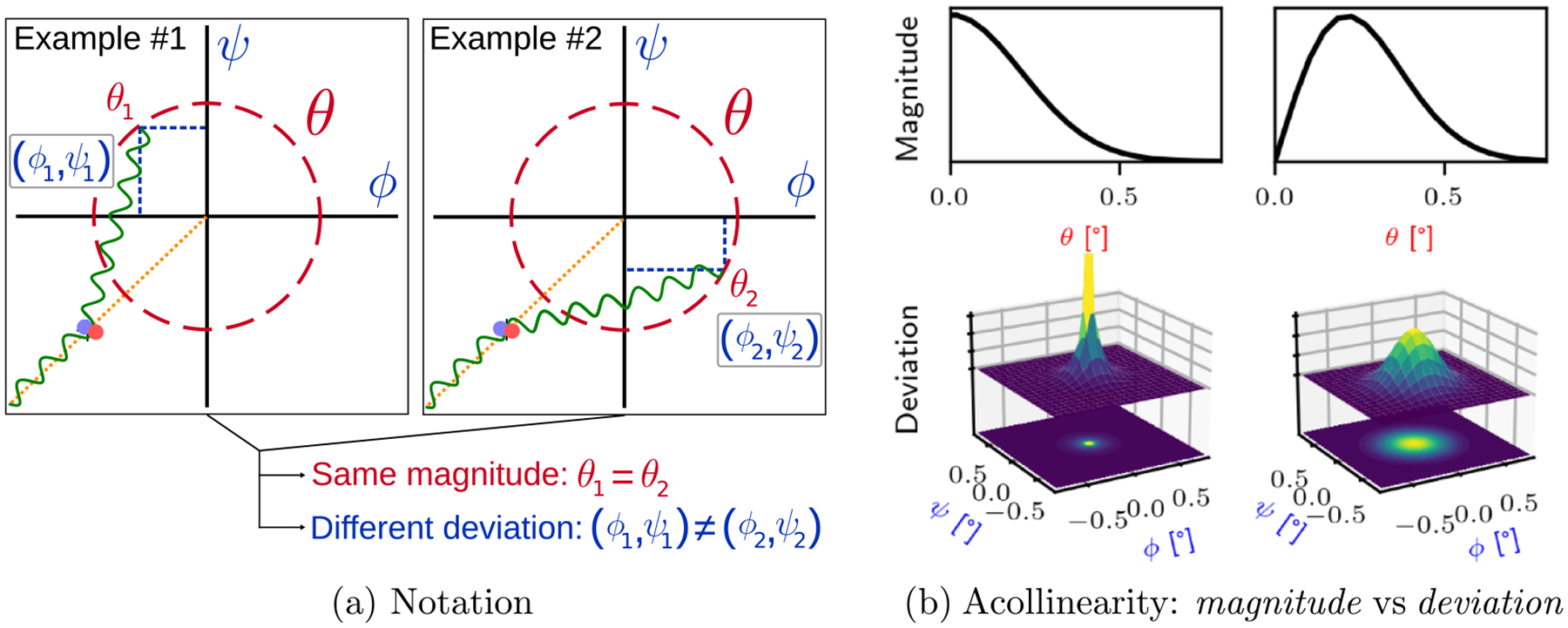
(a) Relation between *magnitude* (θ) and *deviation* (ϕ,ψ) of APA. Two examples of APA are shown with the same *magnitude* (red dashed circle) while their *deviation* is different as indicated by their angular coordinates (blue dashed lines). The dotted orange line shows the collinear case. (b) Comparison of the response function of APA *magnitude*, i.e. f(θ), (top) and *deviation*, i.e. g(ϕ,ψ), (bottom) whether f(θ) or g(ϕ,ψ) is assumed to have a Gaussian profile, respectively left and right.

Let θ be the *magnitude* of the acollinearity and (ϕ,ψ) its *deviation*, in the 3D sphere, with respect to the collinear case (figure 1(a)). Let f(θ) and g(ϕ,ψ) respectively be the response function of θ and (ϕ,ψ). If g(ϕ,ψ) is rotationally invariant (i.e. isotropic), it can be shown that $f(\theta )\approx 2\pi \sqrt{\phi^{2}+\psi^{2}}g(\phi ,\psi )$ for $\left\{(\phi ,\psi )|\theta =\sqrt{\phi^{2}+\psi^{2}}\right\}$. This approximation relies on the assumption that sin(ϕ)≈ϕ and sin(ψ)≈ψ which is reasonable for APA in the PET setting (Shibuya *et al* 2007). Also, g(ϕ,ψ) can be assumed rotationally invariant in PET imaging, since the subject molecules are not oriented in a specific direction (Shibuya *et al* 2007). From here on, when f(θ) or g(ϕ,ψ) is said to have the profile of a statistical distribution (e.g. f(θ) has a Gaussian profile), we mean that it is equivalent, up to a multiplicative constant, to the probability density function of that distribution.

Thus, if f(θ) is assumed to have a Gaussian profile, g(ϕ,ψ) profile would be a 2D Gaussian divided by the norm of its argument (figure 1(b), left). Conversely, if g(ϕ,ψ) is assumed to have a 2D Gaussian profile, f(θ) will have a Rayleigh profile (figure 1(b), right). Note that it is not strictly correct to say ‘g(ϕ,ψ) has a 2D Gaussian profile’ since (ϕ,ψ) is a parameterization on the 3D sphere. In the domain of directional statistics, it would be referred to as a wrapped normal distribution. However, the acollinearity *magnitude* is small enough that the approximation sin(θ)≈θ is valid, as such a 2D Gaussian approximation should be good enough.

The formalism in Shibuya *et al* (2007) relies on using the plane where the two annihilation photons travel. Thus, one could interpret their results as either referring to the acollinearity *magnitude* or *deviation*. In Toussaint *et al* (2023), the formalism of Shibuya *et al* (2007) was extended to use the parameterization (ϕ,ψ), which makes it explicit that it is the acollinearity *deviation* that follows a 2D Gaussian distribution. Thus, APA should be simulated such that the acollinearity *deviation* g(ϕ,ψ) results in a 2D Gaussian profile to be consistent with experimental results. Likewise, any simulation that results in f(θ) having a Gaussian profile would be inconsistent with experimental results. It should be noted that the results of Shibuya *et al* (2007) show that ϕ and ψ are each better represented by double Gaussian distributions than by single Gaussian distributions. We have decided to assume single Gaussian distributions in this work since this is what is implemented in GATE and therefore the observations of this work will only be the result of the difference between the *magnitude* vs. *deviation* interpretations.

In the following, we propose an implementation of acollinearity *deviation* as a 2D Gaussian with a FWHM of *acoValue*. Let $\vec{p}$ be a vector of the 3D unit sphere. Thus, $\vec{p}$ and $-\vec{p}$ would be the directions of annihilation photons for a collinear pair. Without loss of generality, we define APA relative to $\vec{p}$. Let ϕ and ψ each be samples from a Gaussian with a FWHM of *acoValue* and $\vec{u}$ and $\vec{v}$ be orthogonal to $\vec{p}$. Thus, the simulation consists of applying a rotation to $\vec{p}$ of $\theta =\sqrt{\phi^{2}+\psi^{2}}$ in the direction (0,ϕ,ψ) relative to the coordinate system $\left(\vec{p},\vec{u},\vec{v}\right)$. An implementation of APA where its *deviation* follows a 2D Gaussian distribution will be herein dubbed *GaussDev* while it will be dubbed *GaussMag* when it assumes that its *magnitude* follows a (1D) Gaussian distribution.

## 3. Materials and methods

For this study, we had the choice between at least three values of *acoValue*: 0.5° (Colombino *et al* 1964), ≈0.55° (Shibuya *et al* 2007), and 0.58° (GATE’s positron source, from *t19_acollinearity* benchmark). Here, we chose to use the GATE value (i.e. 0.58°) since most of our study was based on the GATE software, allowing GATE users to have a direct interpretation of how the APA misinterpretation has affected their simulations. Also, it was the largest value of the three, thus providing a higher bound on the impact of APA misinterpretation when relying on simulations. This choice means that the theoretical spatial blur of APA in this study is 0.0051×R, where R is the radius of the scanner, rather than 0.0044×R, which is based on the assumption that acollinearity has a FWHM of 0.50° (Moses 2011).

### 3.1. Implementation used to compare both interpretations of APA

To determine which interpretation of APA GATE implements, one needs to look at its source code in the files GateBackToBack.cc and GatePositronAnnihilation.cc. The files indicate that APA is simulated by first sampling the direction of the acollinearity as a uniform distribution and then sampling its *magnitude* as a 1D Gaussian (listing 1). This confirms that the *GaussMag* implementation is used.

**Listing 1.** Original code of GateBackToBack::GenerateVertex() (i.e. *GaussMag*)

G4double dev = CLHEP::RandGauss::shoot(0., acoValue/GateConstants::fwhm_to_sigma); G4double Phi1 = (twopi * G4UniformRand()) / 2.; G4ThreeVector DirectionPhoton(sin(dev) * cos(Phi1), sin(dev) * sin(Phi1), cos(dev));

This can also be confirmed by using the *t19_acollinearity* benchmark provided in the GateBenchmarks repository of the OpenGATE collaboration organization on GitHub^6^. The benchmark simulates both a ^18^F positron source and a back-to-back (BTB) source, the latter with a predefined FWHM for acollinearity that we changed to 0.58° for this study. For both cases, it creates a histogram of the acollinearity *magnitude*, i.e. f(θ). The benchmark was applied to simulations conducted with GATE 9.2 (i.e. *GaussMag* implementation) and with a modified version of GATE 9.2 in which *GaussDev* was implemented as described in the previous section (listing 2).

**Listing 2.** Modification of GateBackToBack::GenerateVertex() in *GaussDev*

G4double phi = CLHEP::RandGauss::shoot(0., acoValue / GateConstants::fwhm_to_sigma); G4double psi = CLHEP::RandGauss::shoot(0., acoValue / GateConstants::fwhm_to_sigma); G4double theta = sqrt(pow(phi, 2.0) + pow(psi, 2.0)); G4ThreeVector DirectionPhoton(sin(theta) * phi / theta, sin(theta) * psi / theta, cos(theta));

### 3.2. Effect on the estimation of spatial resolution via Monte Carlo methods

To provide some insight into the difference in spatial blur induced by the two interpretations of APA, a point source and a Hot Spot phantom were simulated in GATE with both assumptions. BTB sources were used for all simulations. The code used to simulate acollinearity in BTB is equivalent to the one used by GATE for positron sources which is why only the former is shown in this study. In order to avoid the unnecessary complication of scattered events in the analysis, the only physical process activated was *PhotoElectric* with the *StandardModel*. A 40-sided 2D ring PET scanner with a diameter of 81 cm was defined for the simulations. Thus, the theoretical spatial blur of APA is expected to be 2.1 mm. Each side was 64 mm wide and composed of 128 detectors (each 0.5 mm in width, 4 mm axially and negligible depth). All simulations were reconstructed in 2D with the maximum-likelihood expectation-maximization (ML-EM) algorithm using an in-house software that models only the 2D geometric aspect of the scanner. Only true coincidences were kept for the reconstructions.

The point source was placed at [0.15625 mm, 0.15625 mm], which corresponds to the center of one of the image space pixels used for its reconstruction, to avoid the numerical instability often observed at the exact FOV center. Around 100k coincidences were recorded with both interpretations of APA. Prior to reconstruction, a uniform background was added so that the intensity ratio of the point source to the background was less than 110% at convergence to ensure an accurate estimate of the system spatial resolution (Gong *et al* 2016).

The spot sizes in the Hot Spot phantom were 0.5, 0.9, 1.3, 1.7, 2.1, and 2.5 mm. The activity in the phantom was defined so that the expected ratio between spots and background was 4:1. Around 7 M coincidences were recorded with both interpretations of APA. For each simulation, the resolvability of the spots in the optimally reconstructed image, that is, at the iteration with the lowest mean squared error relative to the ground truth, was evaluated using the Rayleigh criterion, as proposed in Hallen *et al* (2020). A semi-automatic implementation of this evaluation can be found in Toussaint and Loignon-Houle (2023).

### 3.3. Impact on the theoretical spatial blur induced in image space

The theoretical spatial blur induced by each interpretation of APA over a range of scanner diameters was also investigated. For simplicity, this analysis only considered a source at the center of a 3D scanner shaped as a sphere and where the only source of blur was APA. In this case, it can be shown that the shape of the spatial response function of the reconstructed source is the same as the angular response function of acollinearity (i.e. g(ϕ,ψ)), scaled by the scanner diameter. In the *GaussDev* implementation, the response function has a Gaussian profile, so the blur can be characterized by its FWHM, i.e. 0.0051×R. For the *GaussMag* implementation, the response function has a vertical asymptote that is ill-defined for FWHM evaluation. However, it has a finite integral, which makes its analysis as a response function possible. We therefore described its effect on spatial resolution as the distance, relative to the center of the response function, for which its integral is equal to the integral spanned by the FWHM of a 3D Gaussian distribution. Note that for a 3D Gaussian, the FWHM spans 29.2% of its integral. Let h(x,y,z) be the (normalized) spatial response function obtained with the *GaussMag* assumption, which is a Gaussian with a FWHM of 0.0051×R divided by the norm of its argument. Thus, we defined the ‘FWHM Equivalent’ metric as $l\in R^{+}|{∭}_{\sqrt{x^{2}+y^{2}+z^{2}}⩽l}h(x,y,z)\text{d}x\text{d}y\text{d}z=0.292$. For comparison, the standard deviation of h(x,y,z) multiplied by 2.355 was also reported. We proceeded by Monte Carlo simulations to estimate these two metrics, given that h(x,y,z) is more complex to study analytically.

## 4. Results

**Figure 2.**
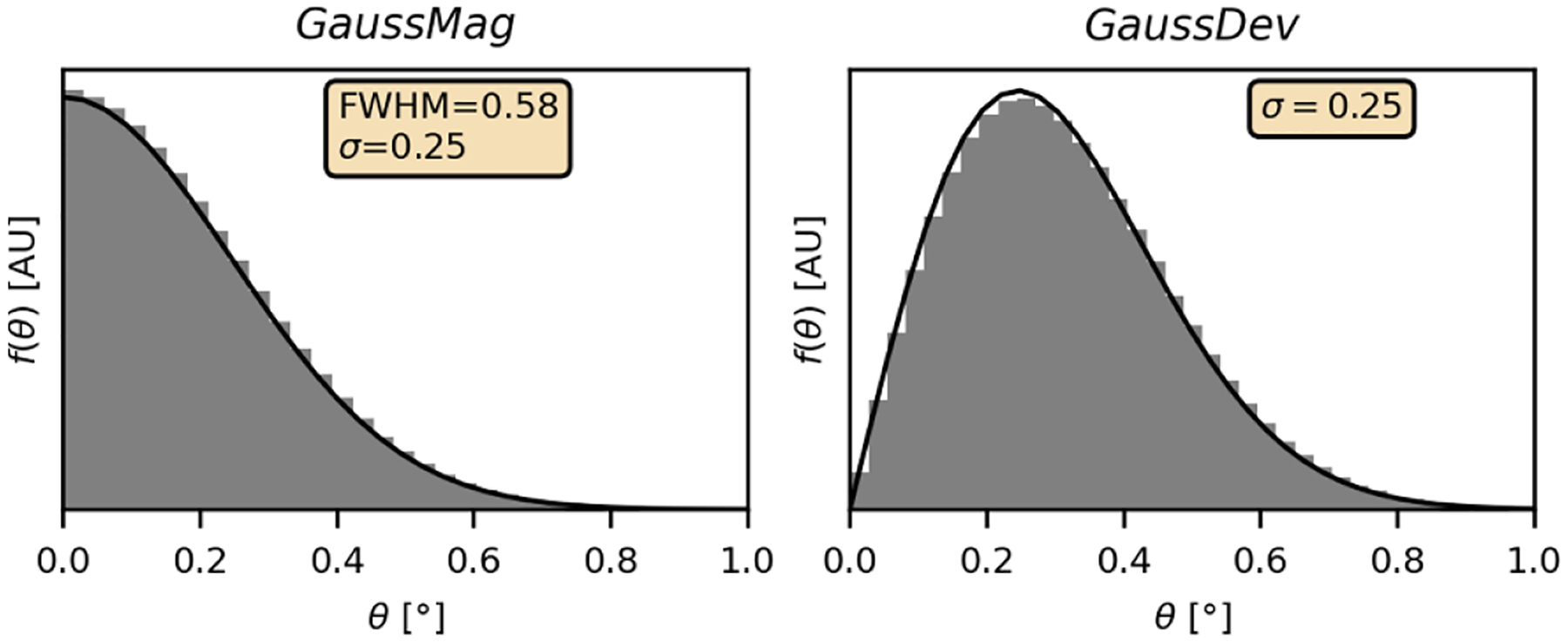
f(θ) obtained by applying the *t19_acollinearity* benchmark on simulations done with the *GaussMag* (left) and *GaussDev* (right) assumptions. A Gaussian function was fitted to the histogram on the left, while a Rayleigh function was fitted to the histogram on the right. The standard deviation of each fit is indicated in inset.

Figure 2 shows the results obtained with the *t19_acollinearity* benchmark. The histogram on the left confirms that f(θ) has a Gaussian profile in GATE, i.e. with the *GaussMag* interpretation which is not consistent with experimental results. On the other hand, the f(θ) obtained with the *GaussDev* interpretation results in a Rayleigh profile, as predicted. This latter result follows from the 2D Gaussian profile of g(ϕ,ψ) (see figure 1(b)), which is in agreement with the experimental results (Colombino *et al* 1965, Shibuya *et al* 2007). Similar histograms were obtained when ^18^F sources were used for the simulation.

**Figure 3.**
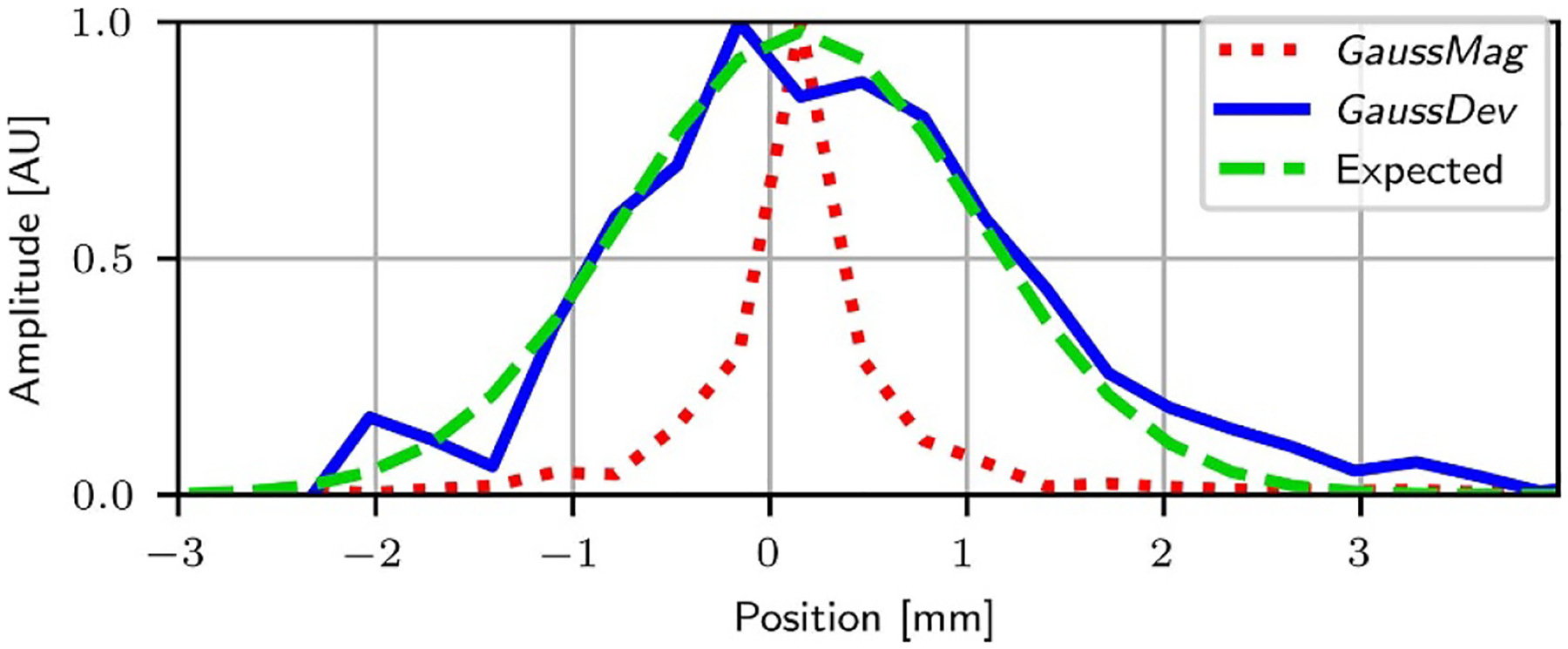
Line profiles of the point source simulated with the *GaussMag* (red) and *GaussDev* (blue) assumptions. Both were reconstructed with 5000 ML-EM iterations to ensure their convergence. The dashed green line is a Gaussian profile with a 2.1 mm FWHM, corresponding to the expected spatial resolution.

**Figure 4.**
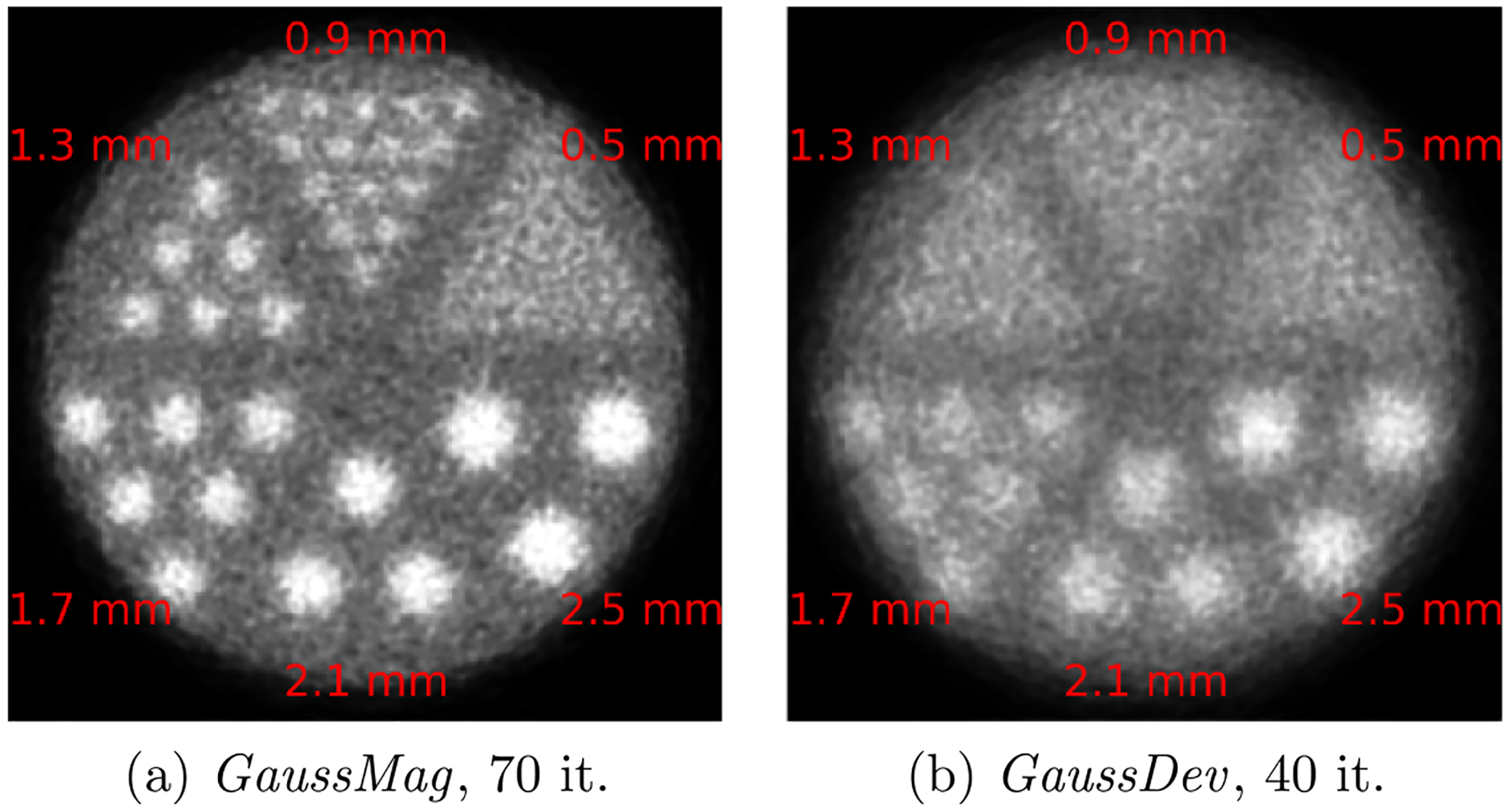
Reconstruction of the Hot Spot phantom with the simulations based on the *GaussMag* (a) and the *GaussDev* (b) assumptions, respectively.

Figures 3 and 4 summarize the results obtained from the simulations of the 2D PET scanner with GATE. As mentioned above, the spatial resolution of this scanner should be limited to 2.1 mm FWHM (0.0051 × 810/2 mm). From figure 3, it can be seen that the blur induced in image space by the *GaussMag* implementation of APA has a cusp shape, which is clearly not a Gaussian profile, and that its FWHM is well below the expected 2.1 mm value. The profile obtained with the *GaussDev* implementation is closer to a Gaussian distribution, with a fitted value of 2.1 mm FWHM. With the Hot Spot phantom, while the small 0.9 mm spots are discriminated with 83.3% resolvability in the image obtained with the *GaussMag* assumption (figure 4(a)), only spots down to 1.7 mm are more realistically discriminated with 44.4% resolvability in the image obtained with the *GaussDev* assumption (figure 4(b)). This simulation highlights how the *GaussMag* implementation, which assumes a Gaussian profile for f(θ), underestimates the effect of APA on spatial resolution.

**Figure 5.**
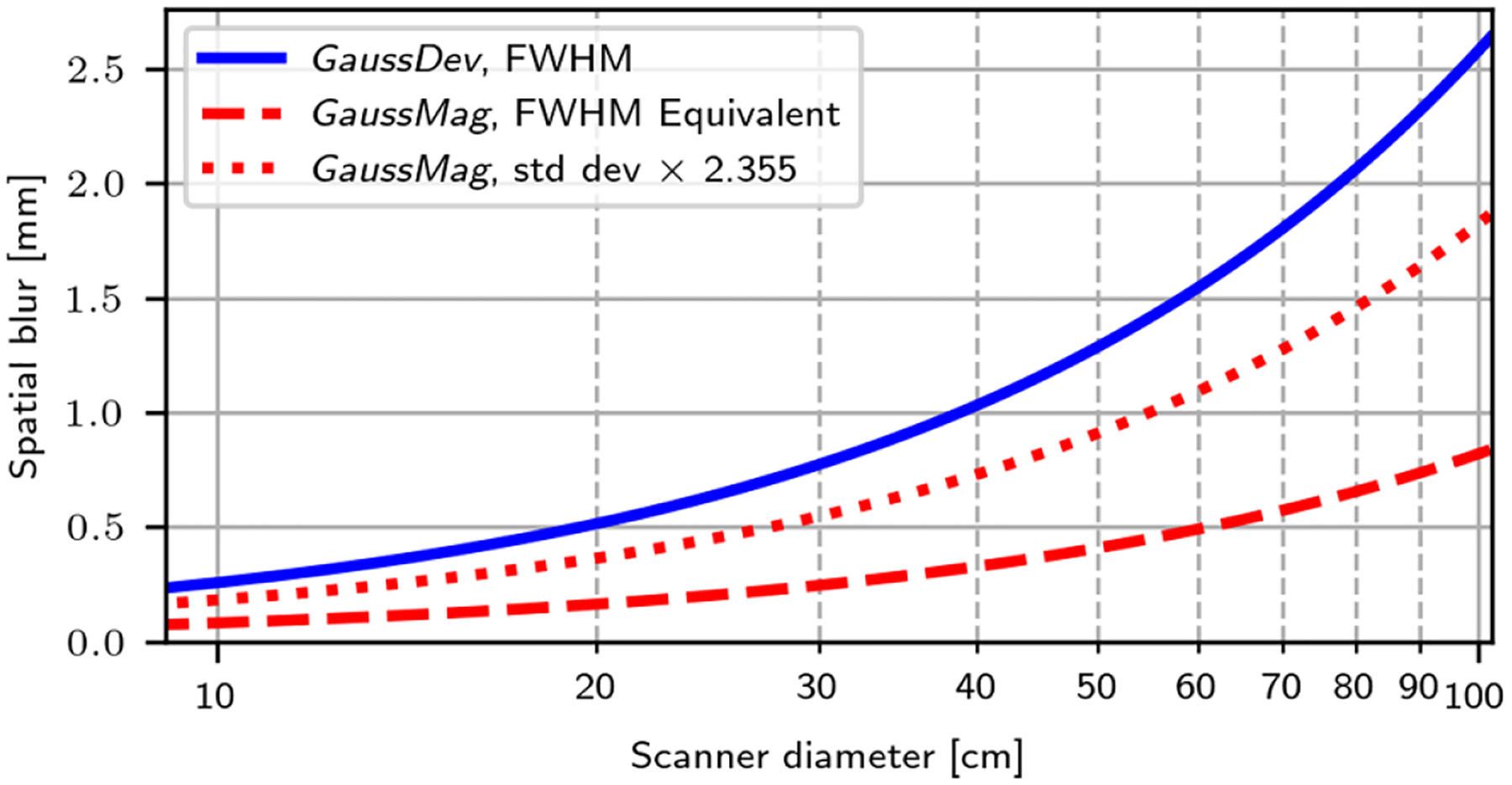
Analytical study of the theoretical spatial blur induced by APA at the center of a PET scanner, as a function of its diameter. For the *GaussDev* implementation (blue curve), the spatial blur is reported as the FWHM of its spatial Gaussian response function. For the *GaussMag* implementation (red curves), the spatial blur is estimated in terms of the FWHM Equivalent metric (dashed line) and as the standard deviation multiplied by 2.355 (dotted line).

Figure 5 shows the theoretical spatial blur due to APA under the *GaussDev* and *GaussMag* assumptions as a function of the diameter for the full range of PET scanners, from a small animal geometry to an approximation of the median length of the 3D lines of response in the EXPLORER total-body scanner (Badawi *et al* 2019). The GaussMag assumption results in a systematic underestimation of the effect of APA on spatial resolution that is particularly significant for whole-body and total-body PET scanners with lines of response exceeding 75 cm in length. For example, the EXPLORER diameter ring is 78.6 cm and it is composed of detectors that are 2.76 mm in width. Therefore, simulations with the *GaussMag* and *GaussDev* assumptions would result in spatial resolutions of ≈1.5 mm and ≈2.4 mm, respectively, when considering only detector size and APA. The gap widens even more for oblique lines of response that can be up to 100 cm in length in a total-body scanner. We also show an approximation of spatial blur obtained by multiplying the standard deviation of the distribution obtained under the *GaussMag* assumption by 2.355, thus assuming that its response function, which has a cusp at its center (bottom left of figure 1(b)), behaves like a Gaussian distribution. For an 81 cm diameter scanner, this would predict a spatial blur of 1.5 mm, which is clearly an overestimation given that most of the 0.9 mm spots in figure 4(a) are resolved. This indicates that the FWHM Equivalent metric is a better predictor of spatial blur than std dev × 2.355 when studying a simulation based on the *GaussMag* assumption.

## 5. Discussion

The notion that APA does not follow the *GaussMag* assumption becomes more apparent when looking at the bottom left of figure 1(b). Indeed, the singularity at 0° suggests that most annihilation photons would undergo almost no acollinearity, which is in contradiction with the known Gaussian blur induced in the image space (Levin and Hoffman 1999). The ambiguity of the interpretation likely arises because the subtle spherical nature of APA may be overlooked when only the *magnitude* of APA is considered.

From the results reported in this analysis, we can conclude that the modeling of APA under the GaussMag assumption, i.e. that f(θ) has a Gaussian profile, is not consistent with experimental results. Figures 3 and 4 show that this inconsistency results in a different shape of blur and an overly optimistic resolvability for a whole-body PET system. The FWHM parameter is ill-defined with the spatial response function resulting from the *GaussMag* assumption, due to its vertical asymptote. The FWHM Equivalent metric was proposed to estimate its effect on spatial resolution. For an 81 cm diameter scanner, it predicts a blur of ≈0.6 mm, which is consistent with what is observed in figure 4(a), where most of the 0.9 mm spots are resolved while the 0.5 mm spots are indistinguishable.

Monte Carlo simulation is currently the standard approach in the initial development phase of new imaging devices to predict imaging performance. Therefore, accurate APA implementation is critical to ensure realistic and not overly optimistic predictions of the achievable spatial resolution of PET scanners.

Since the spatial blur induced by APA is similar to or less than the blur induced by the intrinsic detector resolution of most clinical scanners (e.g. a worst case of 2.5 mm in figure 5), its effect on the overall spatial resolution is usually not readily apparent. This would not be the case in an ultra fast TOF setting, where it has been shown that the effect of the detectors on spatial resolution could be mitigated (Toussaint *et al* 2020b).

The results of this investigation were based on the GATE software. However, it is not the only simulator that models APA under the *GaussMag* misinterpretation. At the time of writing, we found that SimSET (Poon *et al* 2015), gPET (Lai *et al* 2019) and URT-MCS (He *et al* 2023) have also implemented the *GaussMag* hypothesis, and it is likely that other PET simulators use the same assumption. The source code of many simulators, such as PETSIM (Thompson *et al* 1992), PeneloPET (España *et al* 2009), GePEToS (Jan *et al* 2005), GAMOS (Arce *et al* 2014) and SMART-PET (Pfaehler *et al* 2018), is not easily or freely available, so we could not draw any conclusions for them.

While the *GaussDev* implementation seems to provide a more accurate modeling of APA, the fact that some of the 1.7 mm spots are resolvable when the theoretical resolution limit is 2.1 mm requires further investigation. A possible explanation for this behavior is that its response function in the image space may have been modified by using a single ring scanner with detectors of large axial dimension (4 mm was used for faster simulation) while the reconstruction was in 2D. Furthermore, the proposed implementation is an approximation of APA that neglects the spherical nature of the (ϕ,ψ) parameters.

As of version 10.7 (circa 2022), Geant4 (Allison *et al* 2016), the library that GATE uses to define most of its physics, provides the capability of simulating APA in the PET setting. However, this does not mean that GATE based on version 10.7 or later of Geant4 (e.g. GATE 9.2) correctly implements APA. In the following, we examine three ways that positron annihilations can be simulated in GATE, along with the anticipated outcomes. Note that at the time of writing, the latest version of GATE was 9.4.

### Back-to-back sources:

this implementation is directly integrated into GATE, making it independent of Geant4 for acollinearity. Consequently, all simulations using this source, regardless of the GATE version, are contingent upon the incorrect *GaussMag* assumption when acollinearity is enabled.

### PositronAnnihilation^7^ process with positron or ion sources:

this is the recommended process outlined in the GATE documentation to enable APA (OpenGATE Collaboration 2024). The code implemented directly in GATE is used for acollinearity and is therefore independent of Geant4. Thus, all simulations using this approach, regardless of the GATE version, also rely on the incorrect *GaussMag* assumption.

### PhysicsList from Geant4 with positron or ion sources:

if no further modification is made, positron annihilation will be handled by Geant4. Prior to Geant4 version 10.7, simulation of APA in the PET setting was not implemented in Geant4, which resulted in annihilation pairs being collinear. For GATE version 9.1 onward, Geant4 version 10.7 or later is employed. Therefore, this simulation approach can accurately simulate APA in the PET setting. However, this is contingent upon the materials with which the positron interacts having their ‘MeanEnergyPerIonPair’ set to 5 eV (Geant4 Collaboration 2020). Currently, none of the GATE versions do so and they do not offer the option to set it. Consequently, all simulations solely based on PhysicsList and Positron/Ion source will result in annihilation pairs being collinear.

Thus, simulations with any version of GATE will either use the incorrect *GaussMag* implementation or result in collinear annihilation pairs. Consequently, GATE users are currently required to modify the GATE source code, e.g. by setting ‘MeanEnergyPerIonPair’ as stated in Geant4 Collaboration (2020) or by using listing 2 provided in section 3.1 of this Note, to achieve a more accurate simulation of APA.

In the interest of GATE users, the APA response function f(θ) resulting from the combination of three GATE ‘versions’, version 9.2 without modification and with either of the two modifications mentioned above, and five ‘types’ of sources/physics are shown in figure S.1 (supplementary material).

## 6. Conclusion

The literature surrounding the study of APA in PET imaging was revisited to highlight a potential pitfall resulting from a misinterpretation of its physical effect. The ambiguity arises from whether it is the *magnitude* or the *deviation* of APA that follows a Gaussian distribution. We have shown that it was incorrectly implemented as the former in GATE, leading to a significant underestimation of its effect on spatial resolution. An implementation of the latter assumption was proposed and it was shown to be more consistent with the experimental results found in the literature. At least three other PET simulators were found to use the same incorrect implementation of APA, thus a retrospective survey of these (and other) simulators and their results in terms of spatial resolution should be considered.

## Supplementary Material

Supplementary material

Supplementary material for this article is available online

## Data Availability

The data cannot be made publicly available upon publication because no suitable repository exists for hosting data in this field of study. The data that support the findings of this study are available upon reasonable request from the authors.

## Footnotes

6 https://github.com/OpenGATE/GateBenchmarks.

7 Note the missing G4 prefix.
