# Supplementary material for: On the implementation of acollinearity in PET Monte Carlo simulations
Source: Phys Med Biol. Author manuscript; Available in PMC 2026 Mar 23. (PMC13007289; doi:10.1088/1361-6560/ad70f1)
Supplement: Supplementary material [file NIHMS2152305-supplement-Supplementary_material.pdf]

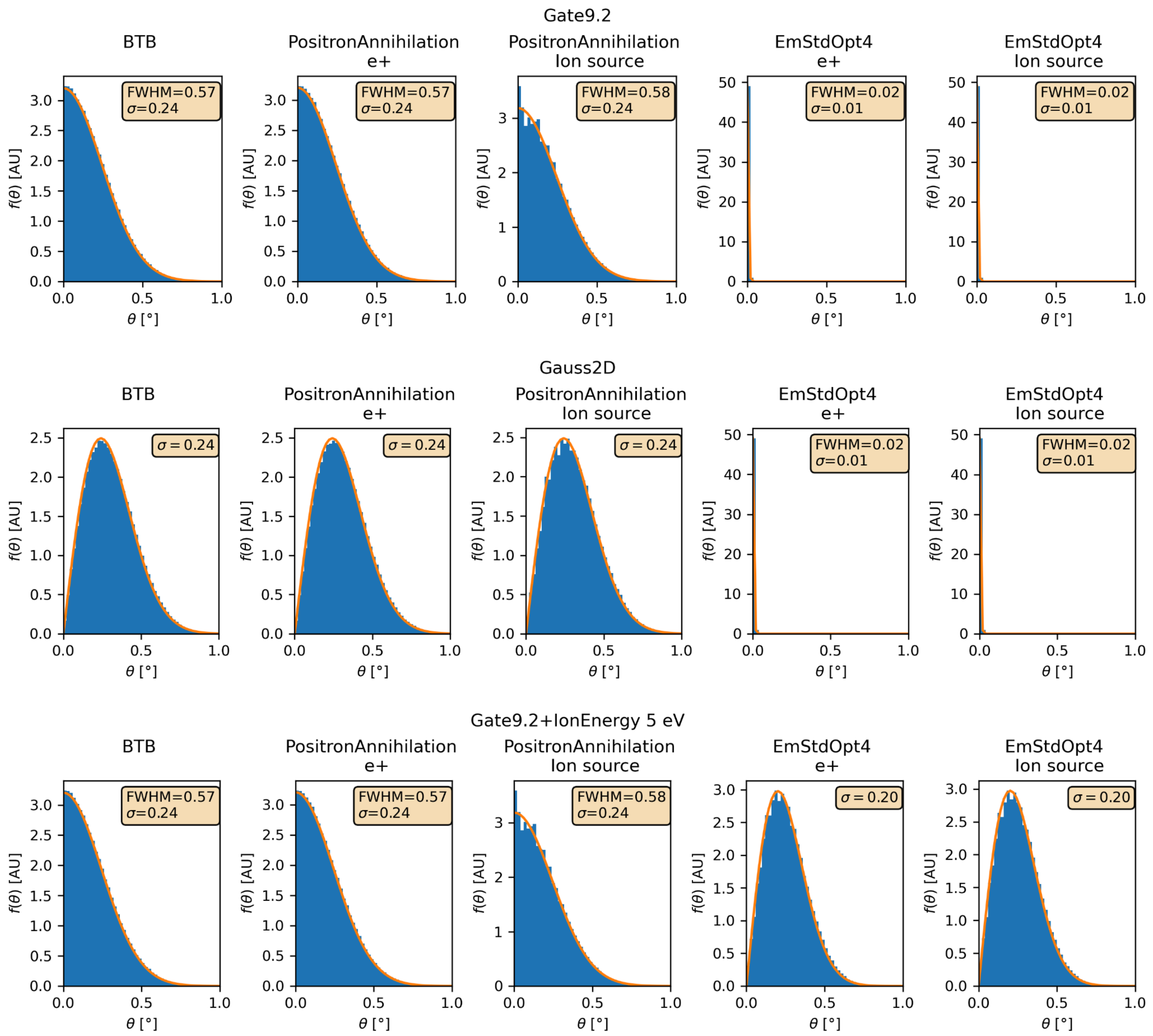

Figure S.1: Comparison of the annihilation photons acollinearity (APA) response function $f(\theta)$ for three “versions” of GATE (rows) and five “types” of sources/physics (columns). The three GATE “versions” are:

- “Gate9.2”: GATE version 9.2 without modification;
- “Gauss2D”: GATE version 9.2 where the code of *Back-to-Back* and *GatePositron* was modified such that acollinearity deviation would result in a 2D Gaussian profile (Listing 2, Section 3.1 of the Note);
- “Gate9.2 + IonEnergy 5 eV”: GATE version 9.2 where the *MeanEnergyPerIonPair* for all materials was hard-coded as 5 eV.

The five “types” of sources/physics are:

- “BTB”: *Back-to-back* with an acollinearity of 0.58° FWHM;
- “PositronAnnihilation --- e+”: *Positron* source using the *GatePositronAnnihilation* class (i.e., not using *PhysicsList*);
- “PositronAnnihilation --- Ion source”: *Ion* source using the *GatePositronAnnihilation* class (i.e., not using *PhysicsList*).
- “EmStdOpt4 --- e+”: *Positron* source using the *PhysicsList G4EmStandard* option 4;
- “EmStdOpt4 --- Ion source”: *Ion* source using the *PhysicsList G4EmStandard* option 4;

Cases that result in a Rayleigh profile demonstrate a proper implementation of APA.
